# Simultaneous recordings of ocular microtremor and microsaccades with a piezoelectric sensor and a video-oculography system

**DOI:** 10.7717/peerj.14

**Published:** 2013-02-12

**Authors:** Michael B. McCamy, Niamh Collins, Jorge Otero-Millan, Mohammed Al-Kalbani, Stephen L. Macknik, Davis Coakley, Xoana G. Troncoso, Gerard Boyle, Vinodh Narayanan, Thomas R. Wolf, Susana Martinez-Conde

**Affiliations:** 1 Department of Neurobiology, Barrow Neurological Institute, USA; 2 School of Mathematical and Statistical Sciences, Arizona State University, USA; 3 Trinity College Dublin, Dublin 2, Ireland; 4 Department of Signal Theory and Communications, University of Vigo, Spain; 5 Department of Neurosurgery, Barrow Neurological Institute, USA; 6 St. James’s Hospital(Mercer’s Institute for Research in Ageing), Ireland; 7 Unité de Neuroscience, Information et Complexité (CNRS-UNIC), France; 8 St James’s Hospital(Medical Physics and Bioengineering Dept.), Ireland; 9 Department of Neurology, Barrow Neurological Institute, USA; 10 Neuro-Ophthalmology Unit, Barrow Neurological Institute, USA; 11 Neuro-Ophthalmology Consultation: Barnett-Dulaney-Perkins Eye Center, USA

**Keywords:** Fixational eye movements, Tremor, Fading, Neural adaptation, Saccadic adaptation

## Abstract

Our eyes are in continuous motion. Even when we attempt to fix our gaze, we produce so called “fixational eye movements”, which include microsaccades, drift, and ocular microtremor (OMT). Microsaccades, the largest and fastest type of fixational eye movement, shift the retinal image from several dozen to several hundred photoreceptors and have equivalent physical characteristics to saccades, only on a smaller scale (Martinez-Conde, Otero-Millan & Macknik, 2013). OMT occurs simultaneously with drift and is the smallest of the fixational eye movements (∼1 photoreceptor width, >0.5 arcmin), with dominant frequencies ranging from 70 Hz to 103 Hz (Martinez-Conde, Macknik & Hubel, 2004). Due to OMT’s small amplitude and high frequency, the most accurate and stringent way to record it is the piezoelectric transduction method. Thus, OMT studies are far rarer than those focusing on microsaccades or drift. Here we conducted simultaneous recordings of OMT and microsaccades with a piezoelectric device and a commercial infrared video tracking system. We set out to determine whether OMT could help to restore perceptually faded targets during attempted fixation, and we also wondered whether the piezoelectric sensor could affect the characteristics of microsaccades. Our results showed that microsaccades, but not OMT, counteracted perceptual fading. We moreover found that the piezoelectric sensor affected microsaccades in a complex way, and that the oculomotor system adjusted to the stress brought on by the sensor by adjusting the magnitudes of microsaccades.

## Introduction

Our eyes are in continuous motion. Saccades, smooth pursuit, reflex eye movements, and vergence eye movements aim our foveas at successive regions of interest. Even during periods of relative fixation, we produce so called “fixational eye movements”, which in human vision include microsaccades, drift, and ocular microtremor (OMT) (Carpenter, 1977; Yarbus, 1967).

Microsaccades, the largest and fastest type of fixational eye movement, shift the retinal image from several dozen to several hundred photoreceptors and have equivalent physical characteristics to saccades, only on a smaller scale (Martinez-Conde, Macknik & Hubel, 2004; Martinez-Conde et al., 2009; Martinez-Conde, Otero-Millan & Macknik, 2013). Drift is a slow (typically < 2°/s) curvy motion, resembling a random walk, that occurs between saccades and/or microsaccades (Engbert & Kliegl, 2004). OMT occurs simultaneously with drift and is the smallest of the fixational eye movements (∼1 photoreceptor width, <0.5 arcmin), with dominant frequencies averaging ∼84 Hz and ranging from 70 Hz to 103 Hz (Bolger et al., 1999; Martinez-Conde, Macknik & Hubel, 2004).

Due to OMT’s small amplitude and high frequency, the most accurate and stringent way to record it is the piezoelectric transduction method introduced by Bengi & Thomas (1968a). The main difficulty with this recording technique is its invasiveness, because the sensor makes direct contact with the eye’s sclera, requiring local anesthesia and holding the eyelid open (i.e. with adhesive tape). Thus, studies focusing on OMT are rare and the perceptual consequences of OMT are virtually unknown. New noncontact methods to measure OMT are in development, but not yet ready for widespread use (Ryle et al., 2009). Such technology may facilitate future studies to uncover OMT’s role in vision.

Here we conducted simultaneous recordings of OMT and microsaccades with a piezoelectric device and a commercial infrared video tracking system (EyeLink II, SR Research). Previous research showed that microsaccades restore visibility to targets that have faded due to adaptation (Martinez-Conde et al., 2006; McCamy et al., 2012; Troncoso, Macknik & Martinez-Conde, 2008). Here we set out to determine whether OMT plays a similar perceptual role. We also tested whether the piezoelectric sensor might affect the mechanical dynamics of microsaccades (i.e. by dampening eye movements during recordings).

Human subjects performed: (1) a Troxler fading experiment (Martinez-Conde et al., 2006; McCamy et al., 2012) to determine the potential contribution of OMT to counteracting fading during fixation, or (2) a simple fixation experiment to determine the effects of the piezoelectric sensor on microsaccade parameters.

We used both the piezoelectric and the video eye tracking systems simultaneously to measure microsaccades, but only the piezoelectric system could measure OMT. Our results showed that microsaccades, but not OMT, counteracted perceptual fading. We moreover found that the sensor affected microsaccade dynamics in a complex way, suggesting that that the oculomotor system adjusted to the mechanical ocular stress brought on by the sensor by adjusting the magnitudes of microsaccades.

This work has been reported elsewhere in abstract form (Otero-Millan et al., 2010).

## Materials and Methods

### Subjects

Eight subjects (5 males, 3 females) with normal or corrected-to-normal vision participated in the experiments. Experiments were carried out under the guidelines of the Barrow Neurological Institute’s Institutional Review Board (protocol number 04BN039). Written informed consent was obtained from each subject.

### Eye movement recordings

#### Video tracker

Depending on the experiment, either binocular or monocular eye position was acquired noninvasively at 500 Hz with an infrared video tracker (EyeLink II, SR Research).

#### Piezoelectric sensor

We measured OMT using the piezoelectric transduction method introduced by Bengi & Thomas (1968a) and refined by Sheahan et al. (1993) and Al-Kalbani et al. (2007). A silicone tipped piezoelectric bimorph was brought into contact with the sclera in the interpalpebral region near the temporal limbus. The voltage generated across the bimorph was amplified by a high input impedance instrumentation amplifier and digitized via a low noise 24-bit analog-to-digital converter (102 dB dynamic range, variable anti-aliasing filter, sampling frequency 2500 Hz) to provide sufficient resolution and dynamic range to capture microsaccades and OMT.

### Experimental setup and procedure

**Figure 1:**
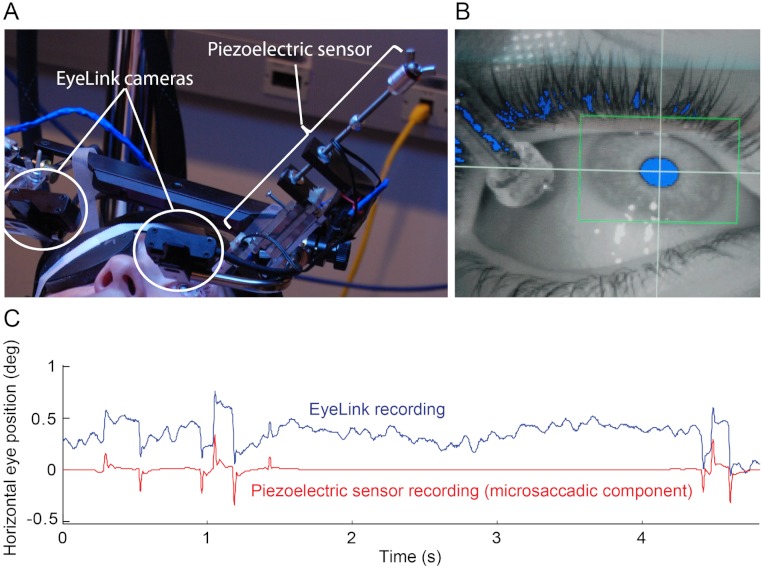
Simultaneous eye movement recording setup. (A) The piezoelectric sensor was mounted to the Eyelink II helmet. (B) Close up of the sensor on the eye in the EyeLink II recording screen. Eyelink II could track the subject’s pupil successfully (blue pixels inside the green box) despite the presence of the sensor. (C) 5 s of raw EyeLink II data (top) and microsaccadic component of the simultaneous piezoelectric recording (bottom). Notice the good correspondence between microsaccades (quick eye position jumps) detected with Eyelink II and the spikes from the microsaccadic component (i.e. a filtered version of the raw data; see Materials and Methods for details) of the piezoelectric recording. The *y*-axis applies to the EyeLink data only.

We mounted the piezoelectric sensor on the EyeLink II helmet to record eye position simultaneously with the two systems (Fig. 1A). Each subject laid supine on a hospital bed, looking up at a horizontally down-facing LCD monitor ∼ 43 cm from the subject. A licensed physician applied a drop of topical anesthetic (proparacaine) to the eye(s) that was/were to have a piezoelectric bimorph. The eyelid(s) of the eye(s) that was/were to have a piezoelectric bimorph was/were then retracted using polyethylene surgical tape. We then let the silicone tipped piezoelectric bimorph rest on the sclera (Fig. 1B) for a maximum of two sequential 40-s trials. Subjects performed one of two experimental tasks (Troxler fading or simple fixation; see Experiments section for details).

### Experiments

#### Fixation experiment

Subjects fixated a small red spot (0.2° radius) on the center of the screen. Before the application of the piezoelectric sensor(s), we conducted a baseline recording of eye movements with EyeLink II. Next, we applied anesthetic drops to the eye(s) that was/were to have a sensor, followed by placement of surgical tape. Then, we lowered the sensor(s) onto the eye(s). We recorded two 40 s trials with the piezoelectric sensor in one or both eyes, followed by a few recovery trials after sensor removal. To account for the potential effects of the topical anesthetic and surgical tape on the eye movement measurements, we recorded some trials in the presence of anesthetic and surgical tape, without the piezoelectric sensor.

#### Troxler fading experiment

This experiment consisted of monocular recordings only (we patched the eye without the piezoelectric sensor). Otherwise the experimental procedure was as above, except that subjects continuously reported whether a visual target was faded/fading (button press) or intensified/intensifying (button release) while fixating the central red spot (Martinez-Conde et al., 2006; McCamy et al., 2012). The visual target (Fig. 4A) was a two-lobe Gabor patch with a peak-to-trough width of 2.5° (Gaussian standard deviations of *x* = 1.5° and *y* = 1°; sine wave period of 5°; sine wave phase of 0), maximum contrast of 40% from peak-to-trough and same average luminance (50%) as the background (Martinez-Conde et al., 2006; McCamy et al., 2012). The Gabor was presented at 0° or 9° of eccentricity measured from the center of the fixation point to the center of the Gabor. The orientation of the Gabor varied randomly between 0° and 360° in each trial, to control for orientation adaptation effects. The position of the Gabor also varied randomly across trials (at one of the eight points of the compass) in the 9° eccentricity condition, to control for possible contrast adaptation effects. After 40 s, the stimuli disappeared and the trial ended. To disregard the potential effect of the initial stimulus onset transient at the start of each trial, we conducted analyses only on data recorded after the first second of the trial.

### Eye movement analyses

#### Video tracker

We identified and removed blink periods as portions of the raw data where pupil information was missing. We also removed portions of data where very fast decreases and increases in pupil area occurred (>50 units/sample, such periods are probably semi-blinks where the pupil is never fully occluded) (Troncoso, Macknik & Martinez-Conde, 2008). We added 200 ms before and after each blink/semi-blink to eliminate the initial and final parts where the pupil was still partially occluded (Troncoso, Macknik & Martinez-Conde, 2008). We identified saccades with a modified version of the algorithm developed by Engbert & Kliegl (Engbert, 2006; Engbert & Kliegl, 2003; Engbert & Mergenthaler, 2006; Laubrock, Engbert & Kliegl, 2005; Rolfs, Laubrock & Kliegl, 2006) with λ = 6 (used for the velocity threshold detection) and a minimum saccadic duration of 6 ms. To reduce the amount of potential noise, we considered only binocular saccades during binocular recordings; that is, saccades with a minimum overlap of one data sample in both eyes (Engbert, 2006; Engbert & Mergenthaler, 2006; Laubrock, Engbert & Kliegl, 2005; Rolfs, Laubrock & Kliegl, 2006). Additionally, in all recording conditions, we imposed a minimum intersaccadic interval of 20 ms, so that potential overshoot corrections might not be categorized as new saccades (Møller et al., 2002). We imposed a maximum microsaccadic magnitude of 2° in both eyes (Beer, Heckel & Greenlee, 2008; Betta & Turatto, 2006; Martinez-Conde et al., 2006; Troncoso et al., 2008). Microsaccade properties (i.e. magnitude, peak velocity) heretofore described were calculated from the EyeLink II data.

#### Piezoelectric sensor

The raw output of a piezoelectric probe on the eye shows the continual, high frequency OMT signal riding on a larger amplitude low frequency signal, consisting of drift and background movement, interspersed with sharp, short, intermittent microsaccades. OMT was defined as vibrations in the 20 Hz to 150 Hz band in the piezoelectric signal output. Simple bandpass filtering of the signal to isolate OMT can cause ringing artifacts in response to microsaccades, whereas cutting out microsaccades causes short periods of data loss. To avoid these issues, a wavelet denoising technique was used to separate the microsaccadic component from the piezoelectric output (Al-Kalbani et al., 2007) (Fig. 1C). In this technique, the OMT and drift components are initially treated as “noise” in the raw piezoelectric signal. The raw signal is transformed to obtain UWT (Undecimated Wavelet Transform) coefficients. The smaller coefficients correspond to signal noise, in this case OMT and drift. The coefficients are thresholded, with coefficients below the threshold set to zero and those above the threshold left unchanged. The threshold levels for denoising were calculated from the universal threshold approach using multiple level rescaling for variance estimation (Luo & Zhang, 2012). An inverse transform is then applied to the thresholded coefficients and the recovered signal contains only the microsaccadic elements. To obtain a microsaccade “free” trace (Fig. 1C), this signal is then subtracted from the original raw piezoelectric signal, leaving only the OMT and drift components of the trace. This trace is then band passed using a 20–150 Hz digital bandpass elliptical digital filter to remove drift and to isolate OMT.

Some piezoelectric sensor data was discarded because technical difficulties with the probe resulted in poor signal, resulting in a total of 15 trials across subjects in the Troxler fading experiment (Fig. 4C).

### Microsaccade and OMT correlations with transitions to visible and invisible percepts

We correlated microsaccade production to the subjects’ perceptual reports, as in McCamy et al. (2012). Briefly, let *X*_*M*_ and *X*_*R*_ be the stochastic processes representing the onsets of microsaccades and intensification reports. For example, if *s*_1_, *s*_2_, …, *s*_*k*_ are the start times of all the microsaccades for a given subject, then *X*_*M*_ for that subject will be given by *X*_*M*_(*t*) = 1 if *t* = *s*_*i*_ for some 1 ≤ *i* ≤ *k*, and *X*_*M*_(*t*) = 0 otherwise; similarly for *X*_*R*_. We obtained correlations of microsaccades with reports of intensification for each subject, using ${\xi }_{M R}(t)={\mathop{\sum }\nolimits }_{n=-\infty }^{n=\infty }{X}_{M}(n+t){X}_{R}(n)$ and then converting it to a rate (similarly for correlations of microsaccades with reports of fading). For each subject, correlations were smoothed using a Savitzky–Golay filter of order 1 and a window size of 151 ms (*X*_*M*_ and *X*_*R*_ were not smoothed). Average correlations are the average of the smoothed correlations (Fig. 4B). OMT correlations with reports of perceptual transitions were obtained in a similar fashion.

### Statistics

To analyze the effect of the piezoelectric sensor on microsaccade magnitude and rate, we conducted separate single-factor repeated measures ANOVAs (one for each dependent variable) with the three measuring times (before sensor, during sensor, and after sensor) as the within-subjects factor. We conducted post hoc comparisons using Tukey HSD tests. To study the effect of the surgical tape and anesthetic on microsaccade magnitude, we used separate two-tailed paired *t*-tests (one for each dependent variable). To analyze the effect of the probe on the microsaccadic peak velocity–magnitude relationship, we used a two-tailed paired *t*-test on the slopes found from the robust linear regressions, for each subject. The significance level was set at α = 0.05. We analyzed the effect of the sensor, tape, and anesthetic on microsaccades using data from the fixation experiment only.

## Results

### Simultaneous recordings and the effects of the piezoelectric sensor on microsaccades

Whereas the piezoelectric sensor can measure both microsaccades and OMT, video systems such as EyeLink II (SR Research) can measure microsaccades accurately, but do not have the resolution necessary to measure OMT (McCamy, Macknik & Martinez-Conde, in press). We extracted the microsaccadic component of the piezoelectric sensor data using a wavelet denoising technique (Al-Kalbani et al., 2007) and we detected the microsaccades from the EyeLink II data using a modified version of Engbert and Kliegl’s algorithm (Engbert & Kliegl, 2003) (Fig. 1C; see Materials and Methods for details). Because the piezoelectric sensor comes in contact with the eye, we wondered whether its presence might affect microsaccade dynamics, for instance by dampening eye movements during simultaneous piezoelectric and video recordings.

We determined the effects of the piezoelectric sensor on microsaccades using data from the *fixation experiment*; see Materials and methods for details. Subjects fixated a central spot and we recorded their eye position binocularly with EyeLink II while applying the piezoelectric sensor in one eye, both eyes, or neither eye.

**Figure 2:**
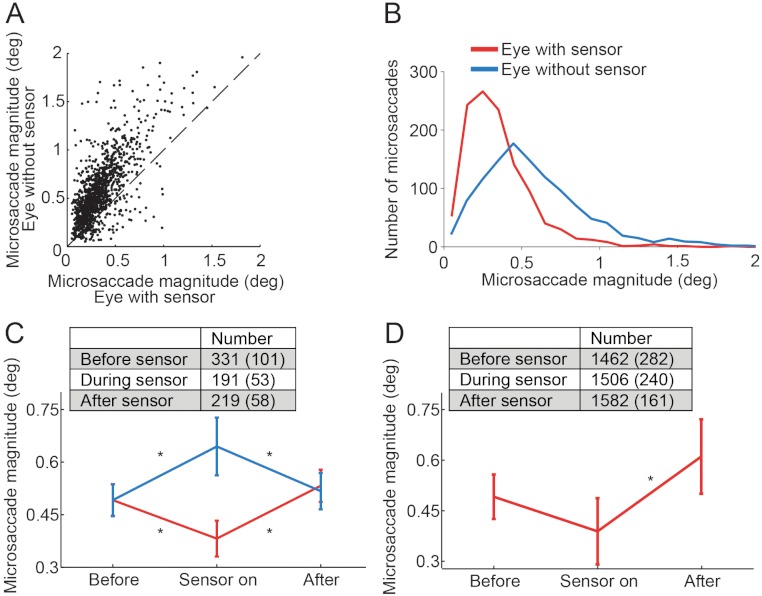
Effects of the piezoelectric sensor on microsaccades. (A) Each dot represents a binocular microsaccade from the *fixation experiment*. Microsaccade magnitude in the eye with the sensor is on the *x*-axis and microsaccade magnitude in the eye without the sensor is on the *y*-axis. Microsaccades were smaller in the eye with the sensor (*n* = 6 subjects). (B) Magnitude distributions of microsaccades from the *fixation experiment*, in the eye with the sensor and in the eye without the sensor (*n* = 6 subjects). (C) Microsaccades in the eye without the sensor were significantly bigger than those prior to sensor application (*F*(2, 10) = 6.49, *p* = 0.016); microsaccades in the eye with the sensor were significantly smaller than those prior to sensor application (*F*(2, 10) = 8.86, *p* = 0.006). Normal microsaccade magnitudes were restored upon sensor removal in both eyes (all Tukey HSD *p*-values > 0.5 for comparisons of Before and After) (*n* = 6 subjects). (D) In the *Troxler fading experiment*, microsaccades in the eye with the sensor also tended to be smaller than those prior to sensor application (though the results did not reach significance, *p* = 0.081). Microsaccades in the eye with the sensor were significantly smaller than after sensor removal (*F*(2, 6) = 12.45, *p* = 0.007) (*n* = 4 subjects). (C, D) Insets indicate the number of microsaccades in each condition. Error bars and numbers in parentheses indicate the s.e.m. across subjects. * Indicates statistical significance using a Tukey HSD posthoc comparison with *p* < 0.05.

Microsaccades occurred as binocular events in every condition, but when a single sensor was placed in one eye, microsaccade magnitudes in the eye with the sensor were significantly smaller than in the eye without the sensor (Figs. 2A–2B and 2C). Further, microsaccades in the eye without the sensor were significantly larger than microsaccades previous to sensor placement, and those in the eye with the sensor were significantly smaller than prior to sensor placement (Fig. 2C). Normal microsaccade magnitudes were restored upon sensor removal (Fig. 2C). Binocular application of the sensor did not alter microsaccade magnitude significantly, but we note that data from this condition were limited to few trials in only two subjects (not shown).

**Figure 3:**
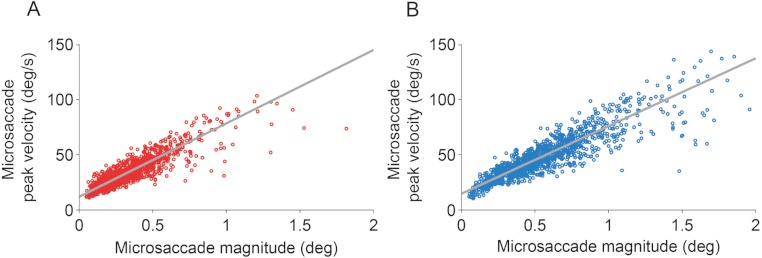
Microsaccadic peak velocity–magnitude relationships. (A, B) Microsaccades from the *fixation experiment* in the eye with the sensor (A) and in the eye without the sensor (B). Plots show data from all subjects for illustrative purposes.

The eye with the sensor had a slightly lower (but not statistically significant, *t*(5) = 1.960, *p* = 0.107) peak velocity–magnitude slope (58 s^−1^ ± 5 s.e.m.) than the eye without the sensor (62 s^−1^ ± 4 s.e.m.) (Fig. 3), suggesting moderately decreased microsaccade velocities in the eye with the sensor.

**Figure 4:**
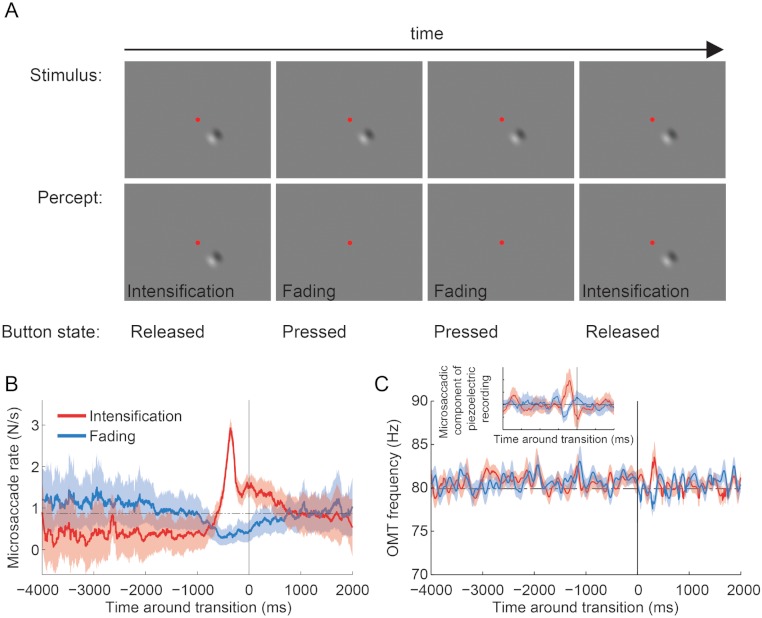
*Troxler fading experiment*: experimental design, and microsaccade rates and OMT frequency relative to reported transitions. (A) Epoch from the *Troxler fading experiment*. Physical stimulus (top row; fixation spot not to scale), subject’s perception of the stimulus (second row), and subject’s report via button press (third row). (B) Average microsaccade rates around reported transitions toward intensification and fading (*n* = 4 subjects). The solid vertical line indicates the reported transitions (*t* = 0). The horizontal dashed line indicates the average microsaccade rate across subjects. The correlation analyses included an average of 1,172 ± 167 transitions to intensification, 1,031 ± 167 transitions to fading, and 5,108 ± 800 microsaccades per subject. Shadows and errors indicate the s.e.m. across subjects (*n* = 4). (C) Average OMT frequency around reported transitions toward intensification and fading. The solid vertical line indicates the reported transitions (*t* = 0). Data collapsed across subjects (*n* = 4) and trials (*n* = 15); see Materials and Methods for details. Shadows indicate the s.e.m. across trials. Inset: Same dataset from main panel. The microsaccadic component from the piezoelectric recording produced comparable correlations with perceptual transitions to those in (B). The correlation analyses included 94 transitions to intensification (main panel and inset), 86 transitions to fading (main panel and inset) and 381 microsaccades (inset only; detected from the corresponding EyeLink data).

The presence of the sensor did not affect microsaccade rates (with sensor: 1.47 microsaccades/s ±  0.36 s.e.m.; without sensor: 1.11 microsaccades/s ± 0.25 s.e.m.; *t*(5) = 2.075, *p* = 0.093).

In the *Troxler fading experiment* (see Materials and Methods for details), applying the sensor to one eye during monocular viewing (i.e. putting a patch on the eye without the sensor) also led to reduced microsaccade magnitudes, as compared to microsaccade magnitudes before sensor application (not reaching statistical significance, *p* = 0.081; *n* = 4 subjects) (Fig. 2D). Microsaccades after sensor removal were significantly larger than those occurring while the sensor was on. Thus, changes in microsaccade magnitude in the eye with the sensor were consistent with those observed in the *fixation experiment*.

Neither taping the eyelids open (with tape: 0.43° ± 0.07 s.e.m.; without tape: 0.46° ± 0.06 s.e.m.; *t*(2) = 1.764, *p* = 0.221) nor applying anesthetic drops (with anesthetic: 0.48° ± 0.06 s.e.m.; without anesthetic: 0.49° ± 0.06 s.e.m.; *t*(2) = 0.475, *p* = 0.682) affected microsaccade magnitudes. Thus, the changes in microsaccade magnitude described above were due to the application of the sensor.

### Microsaccades but not OMT are correlated with perceptual restoration after Troxler fading

Here we set out to quantify the potential role of OMT in restoring faded vision during fixation. Because OMT is not necessarily conjugate (Martinez-Conde, Macknik & Hubel, 2004), we placed a single piezoelectric sensor in either the left or the right eye, and patched the eye without the sensor. Thus, this experiment consisted entirely of monocular recordings.

Four subjects fixated a central spot and continuously reported, via button press, whether an unchanging visual stimulus (a 2-lobe Gabor patch with 40% contrast), which was presented either foveally or peripherally (9°), was faded (or in the process of fading) versus intensified (or in the process of intensifying) (Martinez-Conde et al., 2006; McCamy et al., 2012) (Fig. 4A; see Materials and Methods for details). Microsaccade rates increased before perceptual transitions to intensifying targets and decreased before perceptual transitions to fading targets, in agreement with previous research (Martinez-Conde et al., 2006; McCamy et al., 2012) (Fig. 4B). Foveal and peripheral presentations of the Gabor patch resulted in equivalent modulations of microsaccadic rates before perceptual transitions, also consistent with previous results (McCamy et al., 2012) (data in Fig. 4B are collapsed across both eccentricities). The microsaccades detected with EyeLink II and the microsaccadic component from the piezoelectric recording produced comparable correlations with perceptual transitions (Fig. 4B and Inset from Fig. 4C). OMT frequency was not correlated with either type of perceptual transition (Fig. 4C).

## Discussion

We spend about 80% of our free-viewing time fixating our gaze (Otero-Millan et al., 2008). Vision is moreover suppressed during saccades (Bridgeman & Macknik, 1995; Macknik, Fisher & Bridgeman, 1991; Matin, 1974), and so most visual information acquisition occurs during fixation. Furthermore, in the absence of retinal image motions due to eye movements, visual fading ensues during fixation (Ditchburn, Fender & Mayne, 1959; Drysdale, 1975; Riggs et al., 1953; Sharpe, 1972). Thus, the functions and dynamics of fixational eye movements are important for understanding visual perception.

Here we conducted simultaneous recordings of microsaccades and OMT with a piezoelectric device and a commercial infrared video tracking system to determine whether OMT could help to restore perceptually faded targets during fixation, and whether the placement of a piezoelectric sensor might affect the characteristics of microsaccades. We found that (a) increased microsaccade rates were correlated to the perceptual restoration of faded visual targets, in agreement with previous research, (b) OMT frequency was not correlated with the subjects’ perceptual reports, and (c) the piezolectric sensor affected microsaccade dynamics in a complex way.

### Effects of the piezoelectric sensor on microsaccade dynamics

The oculomotor system adapts to adversities that would, unchecked, impair visual perception. For example, the oculomotor system adjusts its output to account for anatomical change due to growth, damage to the central nervous system or muscular control, and correction of visual refraction from glasses/contacts (Optican, Zee & Chu, 1985; van Donkelaar & Gauthier, 1996). Investigations of the oculomotor system’s adaptive ability have focused on saccadic adaptation, see Pélisson et al. (2010) for a review. In this paradigm, a subject makes saccades to successive cued locations. During the execution of each saccade, the cued location consistently changes (the subject is unaware of this change due to saccadic suppression) to induce a visual error signal at the termination of the saccade. The saccadic system senses this systematic error and slowly adjusts its output (i.e. it recalibrates itself) to compensate for and finally remove the error. The smooth pursuit system can adapt in similar ways as well (van Donkelaar & Gauthier, 1996). Here we encountered another situation where the oculomotor system adjusted its output (i.e. it changed its microsaccade magnitudes) to compensate for the adversity brought on by the piezoelectric sensor.

Lowering the sensor onto the sclera led to (at least) two changes to the state of the eye that could have affected microsaccade magnitude in our experiment: (1) translation and possible rotation of the eye, and (2) dampening of eye movements and deformation of the sclera (upon stabilization after the initial lowering of the sensor onto the eye).

The oculomotor system adjusts its output based on retinal (i.e. visual) and extraretinal (i.e. non-visual) signals (Collins & Wallman, 2012; Pélisson et al., 2010). In the absence of extraretinal signals, image displacements due to eye movements would be indistinguishable from those due to motion in the world; thus retinal signals, by themselves, are not sufficient for accurate visual perception. Extraretinal signals have two possible sources: (a) proprioceptive signals from the extraocular (EOM) muscles and (b) corollary discharge signals from the motor command center. The importance of each type of extraretinal signal is debated, but both are thought to contribute to normal perception (Balslev et al., 2012; Donaldson, 2000; Wang & Pan, 2012; Weir, 2006; Weir, Knox & Dutton, 2000); see Donaldson (2000) for a comprehensive review).

Placement of the piezoelectric sensor on the eye produces a similar scenario to that of the classic “eye press” experiments investigating the contributions of proprioceptive versus corollary discharge signals to perception (Gauthier, Nommay & Vercher, 1990; Rine & Skavenski, 1997; Stark & Bridgeman, 1983). In eye press experiments, one presses on the outer canthus (part of the sclera where the upper eyelid meets the lower one, towards the ear) of the eye while the subject indicates the direction of a target by pointing (Stark & Bridgeman, 1983). The procedure displaces the eye without a corresponding corollary discharge signal, and the experimenter measures the subsequent effects on spatial perception. Experimental conclusions are largely dependent on the assumption that the eye press does not affect the EOM proprioception signals. However, critics have argued that pressing on the outer canthus changes both corollary discharge and proprioceptive signals in a complex and, at the moment, undetermined manner (see Donaldson (2000) for a thorough discussion).Thus, it is unclear whether both translation and rotation occur due to the press and if so, to what extent. It is also unknown whether muscle spindles and palisade endings in the EOMs respond in the same way to passive (i.e. externally imposed) versus active (i.e. internally imposed) movements of the eye. Because of these unknowns, the eye press technique has fallen out of use and the conclusions from studies employing it are debatable (Donaldson, 2000; Rine & Skavenski, 1997). Thus, we can only speculate as to how the inferences from eye press studies relate to our results:

One plausible explanation is that the dampening force of the piezoelectric sensor may have caused microsaccades in the eye with the sensor to be smaller than the oculomotor system intended, thus causing a visual error signal (although subjects did not report any visual errors spontaneously, and received no queries about them) that led to the observed changes in microsaccade magnitude. The mechanical pressure may have also changed the proprioceptive signals of the eye with the sensor. Because classical saccadic adaptation is a gradual process that takes up to 100 trials to complete (i.e. several minutes) (Pélisson et al., 2010), and the “adaptation” we observed was very quick (i.e. the sensor was in the eye for only 80 s) it may be that the sensor affected proprioceptive signals mainly. Proprioceptive signal changes due to dampening – in addition to possible visual error signals in the eye with the sensor – may have resulted in more force and hence innervation in that eye to move as intended. To keep the saccades conjugate (i.e. to obey Hering’s law of equal innervation, which states that during saccades, both eyes receive equal innervation to corresponding muscles (Bahill et al., 1976)), microsaccades in the eye without the sensor may have increased in magnitude (i.e. due to increased innervation), without any damping. In agreement with this possibility, previous studies have found that oculomotor adaptation in one eye translates, at least partially, to the other eye (Albano & Arzola Marrero, 1995; Hopp & Fuchs, 2004; Optican & Robinson, 1980) (albeit oculomotor adaptation can also be disconjugate, see Hopp & Fuchs (2004) and Snow, Hore & Vilis (1985)).

Frens & Geest (2002) found that placement of scleral coil annuli on both eyes of human subjects led to reduced saccadic velocities (by about 5%) and increased saccadic durations (by about 8%). When they placed a coil on one eye only, the effect was still present in both eyes, although it was more variable; thus the authors hypothesized that the effects were non-mechanical, possibly due to an alteration in oculomotor signals. Whereas the OMT sensor has much greater mass than the scleral coil, a similar logic may apply; thus both mechanical and neural factors may have contributed to our observations.

Finally, placement of the sensor resulted in visible deformation of the sclera, which may have affected the shape of the cornea and caused optical blur. It is not clear whether scleral deformation could have affected microsaccade magnitude, but it may have introduced a larger visual error signal and thus contributed to our results.

A definite physiological explanation of the effect of the sensor on microsaccade magnitude remains elusive, due to all the unknown variables detailed above. Future research specifically designed to investigate the effects of changing proprioceptive and corollary discharge signals on microsaccades should provide further insight into how both types of signals combine to give the brain knowledge of gaze position during attempted fixation.

Video oculography systems such as the EyeLink II tracker used here are incapable of measuring OMT (McCamy, Macknik & Martinez-Conde, in press), and so we could not determine the potential effects of the sensor placement on the characteristics of OMT. However, a previous study limited to one subject (Al-Kalbani, 2010), using the same basic probe design as described here, found no significant difference between mean peak OMT frequency measured in both eyes, where the probe mass on one eye was increased by ∼30% (14 g to 18.5 g), compared to the probe used in the other eye. Neither was there any difference observed in the mean RMS OMT amplitude of the eyes with the heavier and lighter probes. Although no apparent relation was found between probe mass and mean RMS amplitude or frequency estimates, there was some greater variability seen in amplitude and frequency within individual records. Confirmation that the probe does not significantly suppress OMT activity would demand repeating the experiment with a “zero” mass probe (i.e. a non-contacting approach). Yet, physiological relevant frequency variations in mean peak OMT frequency clearly do transmit to piezoelectric probes, despite any potential damping, as established in many studies showing OMT frequency changes in different clinical conditions (Bojanic, Simpson & Bolger, 2001; Bolger et al., 2000; Coakley, 1983).

Bengi & Thomas (1968b) also investigated the effect of loading the eye on OMT spectra, and found a progressive reduction in the amplitude of velocity spectral density with increased moment of inertia (via weights on a contact lens) of between 5 and 15 g cm^2^ .

Non-contact laser speckle interferometry offers the possibility of future OMT measurements without interfering with eye dynamics (Al-Kalbani et al., 2009).

#### Implications for the definition of “microsaccade”

Until the 1990s, microsaccades were defined as having amplitudes smaller than 12 min arc. This cut-off value originated in earlier studies finding that the distribution of saccadic sizes during fixation declined sharply around 12 min arc (Collewijn & Kowler, 2008). However, studies conducted in the last two decades found that microsaccade sizes often exceed this value (Martinez-Conde et al., 2009; Otero-Millan et al., 2008; Rolfs, 2009; see also Figs. 2 and 3).

The recent shift to larger microsaccadic magnitudes remains unexplained (Rolfs, 2009). Current and former experimental conditions, including illumination, display type, means of head fixation, and fixation effort, may differ (Collewijn & Kowler, 2008; Rolfs, 2009); another suggestion is that older studies relied on highly trained observers, usually the authors themselves, whereas modern experiments prefer naive participants with little or no fixation experience (Rolfs, 2009).

Another difference is that contemporary human eye-tracking is usually non-invasive, whereas early contact-lens based techniques, such as the optical lever method (Boyce, 1967), required direct and potentially unsafe contact with the eye (McCamy, Macknik & Martinez-Conde, in press). Thus, eye motion hindrance (in addition to altered oculomotor signals, see discussion of Frens & Geest (2002) and Bengi & Thomas (1968b) in the previous section) from the recording apparatus may have led to smaller microsaccades in the early studies. Because non-contact eye trackers leave the eye unencumbered, microsaccades may be free to reach their natural (i.e. larger) amplitude ranges in contemporary studies. Our present data, showing that the application of a monocular piezoelectric sensor decreases (and that sensor removal restores) normal microsaccade magnitudes, are consistent with this possibility (see Martinez-Conde, Otero-Millan & Macknik, 2013, for an in-depth review).

### Effects of microsaccades and OMT on perceptual restoration after Troxler fading

Microsaccade rates increased before perceptual transitions to intensification and decreased before perceptual transitions to fading, indicating that microsaccades counteract Troxler fading, in agreement with previous results (Martinez-Conde et al., 2006; McCamy et al., 2012) (Fig. 4B). However, OMT frequency was not correlated with perceptual restoration of faded targets in the present conditions (Fig. 4C).

It is important to note that the limited amount of data available from the piezoelectric recordings (Fig. 4C Inset) generated microsaccade correlations with perceptual transitions that were comparable to those obtained with the full video tracker recordings dataset (Fig. 4B); thus it seems unlikely that the lack of a correlation between OMT frequency and perception was due to insufficient data (because the same amount of data did render a correlation for microsaccades). Yet, our results do not completely rule out a contribution of OMT to combating visual fading. For instance, differences in OMT frequency or amplitude across subjects might cancel out in the average, thereby diminishing a potential correlation with perceptual transitions in our analyses.

In addition, OMT may improve or enhance other visual functions, such as signal detection (Funke, Kerscher & Wörgötter, 2007) or visual acuity (Hennig et al., 2002; Zozor, Amblard & Duchêne, 2009). Future research should investigate the potential effects of OMT in preventing and restoring faded targets of varied spatial frequencies, eccentricities and sizes, as well as its possible role in other perceptual phenomena.
